# Preferences for telehealth: A qualitative study with people accessing a new mental health service

**DOI:** 10.1177/20552076231211083

**Published:** 2023-10-31

**Authors:** Anne Honey, Monique Hines, Rebecca Barton, Bridget Berry, John Gilroy, Helen Glover, Nicola Hancock, Shifra Waks, Karen Wells

**Affiliations:** 1 522555 Faculty of Medicine and Health, The University of Sydney, Sydney, Australia; 2 Enlightened Consultants, Brisbane, Qld, Australia

**Keywords:** Mental health, COVID-19, telehealth, lived experience perspectives, community mental health support

## Abstract

**Objectives:**

To examine preferences for telehealth versus in-person services for people who sought mental health support from an unfamiliar service during the COVID-19 pandemic and to identify the factors that influenced these preferences.

**Methods:**

Data are drawn from semi-structured interviews with 45 participants (32 people who accessed mental health services, 7 informal support people, and 6 people who accessed services themselves as well as identifying as informal supports). Data relating to experiences of telehealth, comparisons with in-person services and preferences were coded inductively and analysed using qualitative content analysis.

**Results:**

Just over half of the participants in our sample preferred telehealth or at least regarded it as a suitable option. Those who preferred telehealth were more likely to have had direct experience, particularly via videoconferencing, as part of their access to this new mental health service. Reasons for preferring in-person services included belief in the superiority of interpersonal communication in these settings, compatibility with personal communication style and discomfort with technology. Those preferring telehealth cited its convenience, elimination of the need to travel for services, the comfort and safety afforded by accessing services at home and the ability to communicate more openly.

**Conclusions:**

Hybrid models of care which harness the unique benefits of both in-person and remote service modalities appear to have a legitimate place in models of mental health care outside of pandemic situations. These results illuminate the potential of telehealth services when engaging with people seeking mental health help for the first time and in situations where existing relationships with service providers have not yet been established.

## Background

Telehealth delivery of mental health services is a rapidly evolving field, and historically has been proposed as a promising way of addressing unmet needs for clinical services, particularly for addressing service inequities in rural and remote areas.^1^ More recently, telehealth has rapidly emerged as a method of delivering real-time services in the face of the COVID-19 pandemic and public health restrictions on in-person contact.^2^ One study in the United States found that the percentage of claims for mental services provided via telehealth increased from a pre-pandemic level of 1% to a peak of 53–59% in April 2020.^3^ Rates of claims for telehealth-delivered mental health services later plateaued to a level of approximately 40% of all claims by the end of 2021. This pattern contrasted with that of telehealth claims for general physical health conditions, which saw a much lower increase in April 2020 and reducing to only 5% of all claims by the end of 2021.^3^

Sustained uptake of telehealth delivery of mental health services has similarly been observed in Australia, with one study finding that by the end of 2021, 15–20% of mental health services were delivered by videoconference and 9–15% by telephone, rising from a pre-pandemic levels of approximately 1–2% of all mental health services.^4^ Such research suggests that changes in telehealth uptake for mental health concerns are likely to be sustained into the future.^3^

Research indicates that mental health services delivered via telehealth are as effective as in-person services for a range of mental health concerns^5–9^ and do not compromise the quality of the clinician–client therapeutic relationship.^10^ Telehealth services are highly acceptable to service users^11^ and mental health professionals alike.^12^ For instance, a study of over 3000 service users found that over 80% reported a good or excellent experience with telepsychiatry.^11^ Providers, whilst generally supportive of telehealth tends express less positive views of telehealth, citing concerns regarding the safety, effectiveness and impact on therapeutic relationship with service users.^12^ Despite the obvious promise of telehealth, uptake and integration into routine care prior to the onset of COVID-19 had been slow.^13^

In Australia, as in other parts of the world, COVID-19 triggered an escalation in the use of telehealth by mental health service providers.^3,14^ Increased rates of mental health distress associated with COVID-19 were experienced^15^ and a range of strategies to improve access to needed psychological support were implemented. For instance, the Australian Government provided additional subsidies for mental health treatments, including telehealth, via the medicare benefits schedule (MBS). It is possible that these systemic changes in policy and funding may have helped to support positive clinician attitudes towards telehealth. Evidence suggests that clinicians’ acceptance increases with use and familiarity with telehealth.^12,16^

Since clinicians are often ‘gatekeepers of implementation’,^17^ most research on telehealth implementation has focused on understanding the perspective of clinicians and service providers. Much less is known about how people who access mental health services experience telehealth and the factors influencing their preferences for future service modalities. There is increasing recognition of the importance of understanding and incorporating the perspectives of people who access services in service planning and program development.^18^ As early telehealth experiences have a critical influence on people's attitudes towards future telehealth engagement,^19^ it is important to understand how these are experienced and how this may shape ongoing service modality preferences.

Some evidence suggests that although people expressed divergent opinions and preferences about telehealth and in-person service delivery, telehealth is highly acceptable to many people who access mental health services.^11,20,21^ Many report that they are likely to access telehealth services in some form in the future.^22,23^ However, this evidence is primarily drawn from studies utilising survey methodology, a format that is not well-suited to deeper exploration of the experiences and perspectives of those who access services.^22^ There are few qualitative investigations of people's perspectives of telehealth that employ interview methodology. Those that do have focused specifically on the experience of people who were already engaged with in-person services prior to adoption of telehealth,^24^ on the experiences of people diagnosed with moderate to severe enduring and complex mental health conditions,^21^ or have not included the perspectives of those attempting to access mental health services for the first time.^25^ This is particularly important given the low proportion of people living with mental ill-health who access mental health support.^26^

The current study also adds to existing knowledge by reporting data from people within Australia accessing a new mental health service and from some who accessed mental health support for the first time. As pre-existing relationships established in-person between clinicians and their clients may influence the subsequent acceptability of telehealth services^25^ it is important to understand how people new to a particular service experience telehealth. Tele-mental health services may hold particular benefit within an Australian context by overcoming intractable challenges of delivering specialist services to populations dispersed across large distances, so further exploration of Australians’ preferences for treatment modalities could help ensure that future services are designed in a way that best meet people's needs.^21^ The objectives of this study are: (a) to use secondary data to examine the preferences for telehealth versus in-person services for people who sought mental health support from an unfamiliar mental health service and (b) to identify the factors that influenced these preferences.

## Methods

### Study design

This paper reports on a qualitative content analysis^27^ of data relating to preferences for in-person and telehealth services, drawn from in-depth interviews conducted as part of a larger evaluation of a new mental health service, HeadtoHelp, in Victoria, Australia. Qualitative content analysis provides a systematic way to identify and describe meanings within texts, with consideration given to both the manifest and latent content of texts, including contextual information that shapes meanings.^27^ As such, we draw from a constructivist/interpretivist paradigm, where meanings are assumed to be subjective, context-dependent and complex, requiring interpretation, rather than being reflected in a single, objective reality.^27^ This research approach is well-suited to exploratory investigations of topics or phenomena about which little is currently known.^27^

The research team included lived-experience researchers who had extensive input into all stages of the research, including research design, data collection, and data analysis and interpretation. Ethical approval was granted by The University of Sydney Human Research Ethics Committee (Project #2021/222). We followed the Standards for Reporting Qualitative Research recommendations.^28^

### Context

HeadtoHelp was introduced in September 2020 in the state of Victoria, in response to extensive stay-at-home lockdown restrictions. Funded by the Australian Government, it represented a new model of care in which people contacted a single, central intake line and, following assessment of their needs, were connected to existing services or received care in-person or via telehealth at one of 15 new HeadtoHelp hubs. Data for this evaluation were collected between May and November 2021. Three lockdown periods occurred during the data collection period, with the most significant spanning 77 days (5 August–21 October 2021). During these periods, people were encouraged to access mental health care via telehealth, but in-person, face-to-face services were permitted in cases of urgent care where telehealth was not deemed to be clinically appropriate.

### Sampling and recruitment

Potential participants were all people who had completed a HeadtoHelp intake assessment between September 2020 and August 2021 and provided consent to be contacted (excluding those requiring acute services) as well as informal support people who had contacted HeadtoHelp in relation to mental health issues being experienced by a family member or friend. Potential participants were invited to express their interest in participating in an interview with the research team. A total of 3911 invitations were sent via text message from HeadtoHelp in May 2021 (for those accessing HeadtoHelp between September 2020 and March 2021) and September 2021 (for those accessing HeadtoHelp between April and August 2021). This response rate was in line with the response to other service participant experience survey requests^29^ even though the requirement in the current study was greater (being a request for an interview rather than simply survey completion), indicating a relatively high level of interest.

Invitations contained a weblink to an online expression of interest (EOI) form, a secure data capture tool with survey function. Those interested submitted an EOI directly to the independent researchers either via the online form or via phone or email, where a researcher entered information supplied by the interested person into the online form. This process ensured that HeadtoHelp did not know which service users participated in the research, and researchers did not have access to contact details of service users not volunteering to participate in the research. A total of 386 EOIs were received.

**Figure 1.**
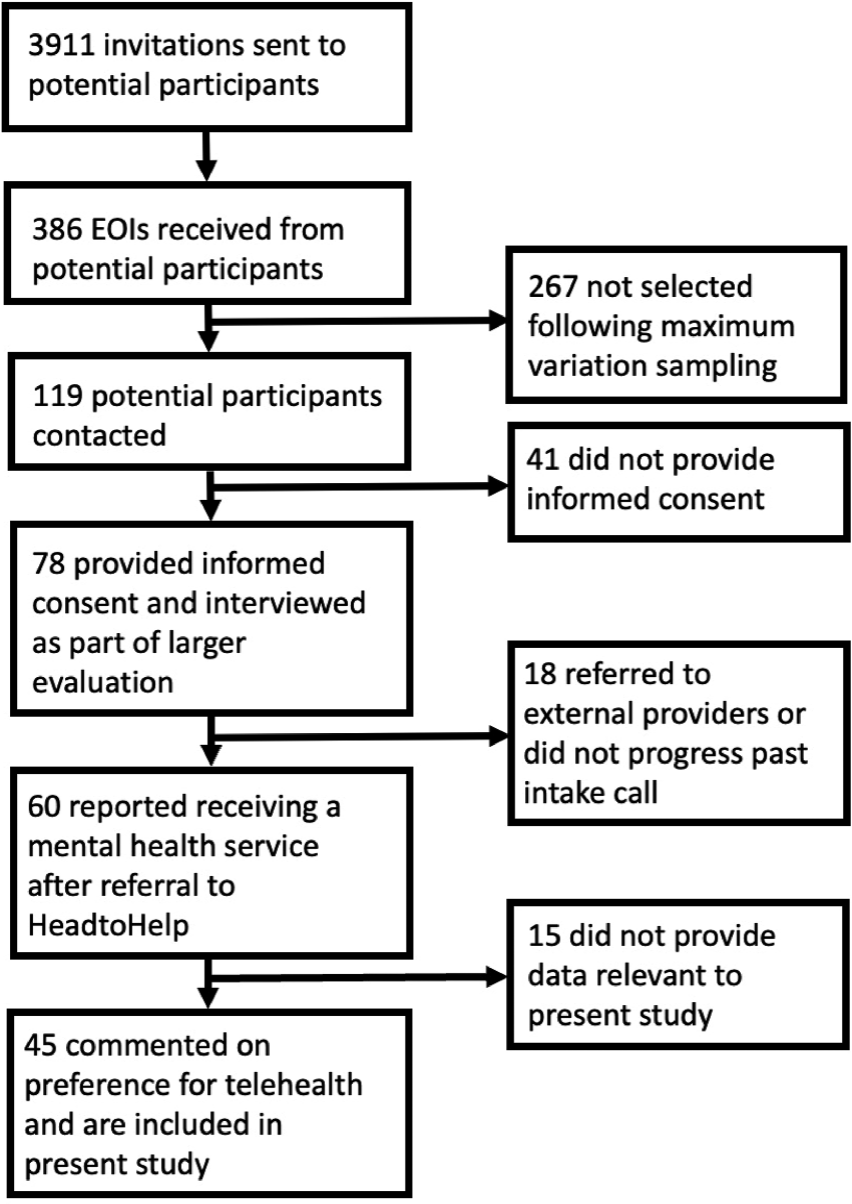
Flow diagram of included participants.

Maximum variation sampling^30,31^ was then used to select participants to obtain diversity in characteristics ascertained through the EOI, including age, gender, cultural and linguistic background, area type, service received, and service satisfaction. Selected participants were contacted by researchers via telephone, provided with an information sheet and given an opportunity to have any questions answered. 119 potential participants were contacted via telephone call or text message, of whom 78 provided written or verbal informed consent and were interviewed. At this point, data saturation was reached; that is, additional interviews were not generating new concepts and existing categories were well understood. Of the 78 participants, 60 reported receiving a mental health service. The remainder were referred to another provider or did not progress beyond the initial call. Forty-five people who received services commented on their preferred service delivery mode. It is the experiences of this sub-group of participants that we are reporting on in this paper. Figure 1 provides a summary of participant recruitment and selection for this secondary analysis. Participants who completed an in-depth interview received a $25 gift voucher as compensation for their time.

### Data collection

In-depth, semi-structured interviews were conducted to explore participants’ unique perspectives and experiences.^32^ All but one interview was conducted by researchers with a lived experience of mental health issues (SW, HG, KW). All (RB, SW, HG, KW) had experience in conducting in-depth interviews with people with mental health issues. None had any prior relationship with participants. Interviews were held via telephone and ranged in length from 16–110 min (mean = 40 min), depending on how much the participant wished to share. Each interview was audio-recorded with participants’ permission and transcribed verbatim for detailed analysis. An interview guide was developed to reflect topics of interest to the government department responsible for the service, specifically: (a) perspectives on the service user journey including accessing HeadtoHelp, receiving services and service discharge; (b) the contexts under which people sought and received HeadtoHelp services and (c) the impact of services on their daily lives. This interview guide was used flexibly to focus on issues of concern to participants and to avoid potential distress. People were asked broad, open-ended questions about their experiences of accessing and receiving services from HeadtoHelp, such as ‘What would you say is the best thing about HeadtoHelp?’ and ‘Are there things you didn’t like or found frustrating about HeadtoHelp?’ The interview guide did not contain any questions that specifically sought information about participants’ preferences for telehealth. However, this topic was often mentioned by participants in the context of describing where their services took place and their perspectives on how helpful these services were. The conversational manner of the interviews meant that probes were often used to clarify the mode of service received (i.e. in-person or telehealth) and how people felt about that, if relevant to the interview and not spontaneously mentioned.

### Data analysis

Data were analysed as soon as possible after each interview to allow researchers to explore aspects or topics raised by earlier participants in more detail in subsequent interviews and to inform decisions about when data saturation had been reached. Transcripts were de-identified, labels assigned to each participant, and reviewed by members of the research team for accuracy. Interview data were analysed using constant comparative analysis, a systematic and well-regarded qualitative analysis method^33,34^ often associated with the grounded theory method but equally useful for qualitative content analysis. NVivo qualitative data analysis software was used to facilitate data management and coding. Researchers conducted close readings of transcripts and then inductively coded the text line-by-line, with categories and themes drawn directly to reflect the raw data. Coding is the process of defining what is happening in the data by giving a short name to small parts of the transcripts that represent a particular idea. Each new chunk of data was compared to previous data and existing codes to determine whether the underlying concepts were the same or different. If different, a new code was generated. Codes were then compared to each other to group similar codes together into higher level categories and to identify the relationships between the codes. This type of analysis ensures concepts that emerge are grounded in the data rather than influenced by pre-existing ideas, increasing credibility of the findings by ensuring that they represent participants’ responses.

The research team was a diverse mix of researchers with expertise in mental health lived experience, mental health service evaluation, telehealth and cultural diversity, each bringing their own perspective to the analysis. Ongoing reflexive discussions in regular meetings within the research team throughout the study ensured consensus on the interpretation of data. Having researchers with lived experience of mental health issues provided rigour, helped ensure that the analyses faithfully represented participants’ views and added depth and richness to the interpretation of data.

This paper reports on the codes related to people's experiences and preferences around in-person and telehealth services. Detailed secondary coding of this data was conducted by the second author. To enhance rigour, the first author then reviewed the raw data and audit trail, compared assigned codes and the authors reached consensus on the analysis framework. The framework was discussed with the rest of the team to ensure it fit with their understanding of the data and to enable questioning and refinement of codes and categories. Frequency counts of codes were calculated and compared across participant groups in order to generate insights supplementary to the main qualitative analysis.^35^ However, it should be noted that these do not necessarily reflect the actual prevalence or level of importance of each theme.^35^ With qualitative interviews, a participant not mentioning a concept is not taken to mean that they did not experience it.

## Results

The demographic and program involvement characteristics of the 45 study participants who received services and commented on their preferred service delivery mode are provided in Table 1. All but one participant who accessed telehealth services did so on an individual basis from their own home. The one exception was a participant who had accessed one session of couples therapy with their partner, followed by individual sessions.

**Table 1.** Demographic and access data.

Characteristic	People who access MH services (n = 32)	Providers of informal support (n = 7)	Both accessing MH services and providing informal support (n = 6)
Age: mean	39.8 years	42.0 years	52.3 years
Age group			
Under 18 years	0	0	0
18–24 years	6	0	0
25–34 years	8	0	1
35–44 years	10	5	0
45–54 years	3	2	4
55–64 years	1	0	0
65+ years	4	0	1
Gender			
Female	19	6	5
Male	10	0	1
Gender non-binary	3	0	0
Prefer not to say	0	1	0
Diversity			
Aboriginal or Torres Strait Islander background	7	0	0
Speaks a language other than English at home^a^	10	0	0
Identifies with a culture other than ‘Australian’	12	0	0
Identifies as part of the LGBTQI+ community	7	0	0
Previous contact with mental health services			
First time approaching mental health services	10	1	2
Previously used mental health services	22	6	4
Area of residence			
Melbourne	16	3	3
A regional city (e.g. Geelong, Mildura)	6	2	2
A rural or remote area	10	2	1
Relationship of support person to loved one	n/a		
Parent		6	3
Partner/spouse		0	3
Sibling		0	0
Another relative		0	0
Friend		1	0
Age of support person's loved one: mean	n/a	13.5 years	34.8 years
Age group			
Under 18 years		4	0
18–24 years		2	2
25–34 years		0	1
35–44 years		0	0
45–54 years		0	2
55–64 years		0	0
65+ years		0	0
Missing		1	1

^a^ Languages included Sinhala, Somali, Bengali, Thai, Cantonese, Filipino dialect, Arabic, Spanish, Serbian, Italian and Turkish.

Quotations below are attributed by participant number and identified as people who accessed mental health services for themselves (GROUP A), informal support people who had assisted their loved ones to access services (GROUP B) and those who identified as having both roles (GROUP AB).

**Figure 2.**
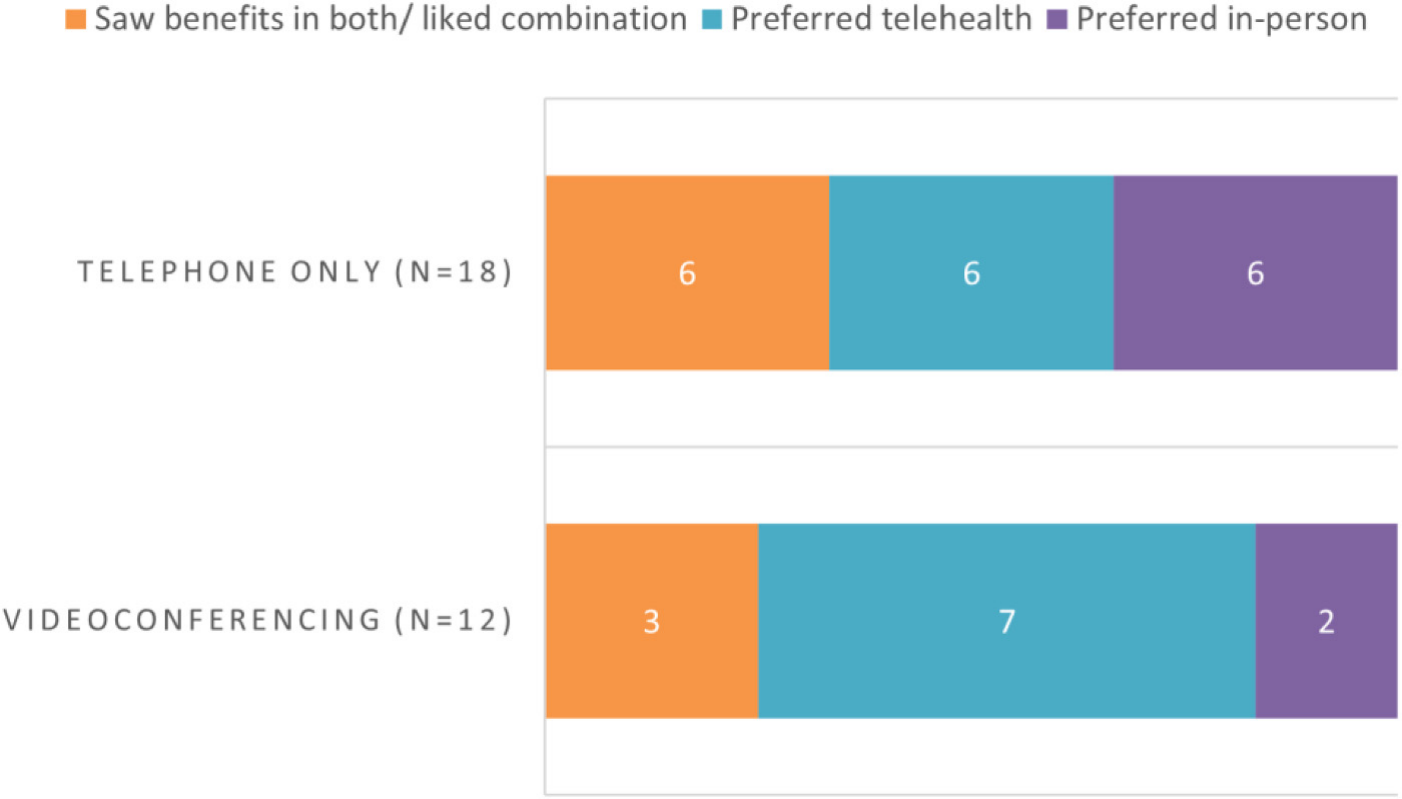
Preference for telehealth or in-person services by mode of telehealth experienced.

Of the 45 people in our sample, 21 participants said that they preferred in-person services, 16 said that they preferred telehealth services and 8 described merits of both modes of service delivery and appreciated a combination. Figure 2 describes participants’ preferences for in-person or telehealth services according to the service mode received (in-person only (*n* = 12), telehealth only (*n* = 17), both in-person and telehealth (*n* = 16)). Those with direct experience of telehealth services were more likely to express preference for telehealth services in the future.

**Figure 3.**
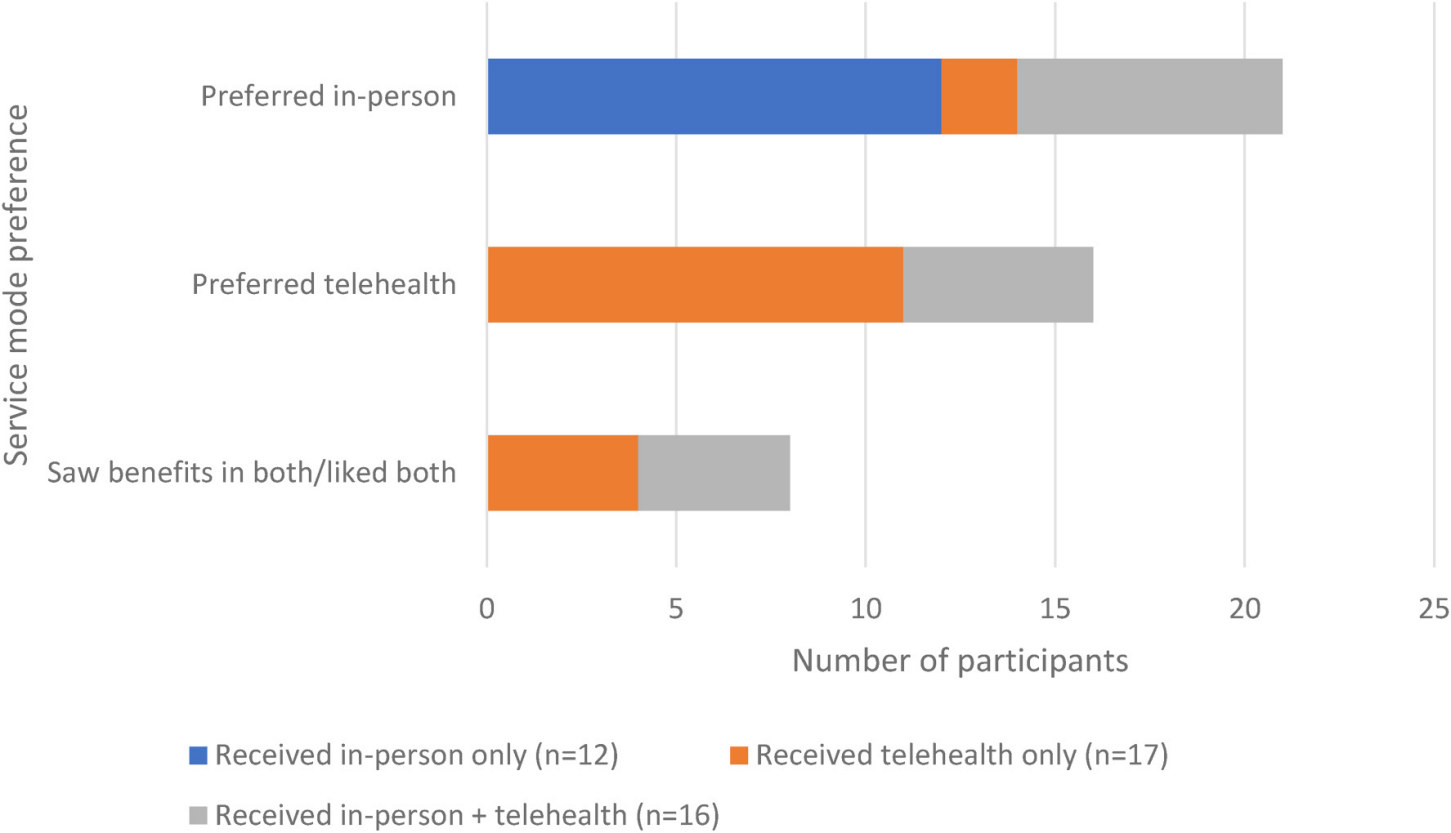
Preference for in-person or telehealth service by service received.

Figure 3 presents data on the preferences for in-person or telehealth services for the subset of participants who received telehealth services. Results are presented according to the type of telehealth services they received, either via telephone or videoconferencing. While numbers are small, a higher proportion of people who had experienced videoconferencing compared to telephone only preferred it or liked a combination.

No influence of participants’ age, gender or cultural background on their expressed preferences for service modality was discerned.

### Reasons for preferred service delivery mode

#### Preference for in-person services

Participants who said they preferred in-person services described a range of reasons. The most common was the belief that personal human contact and connection was superior in in-person settings (n = 16). This was often described as related to personality and general preference, with some expressing the belief that their communication style did not suit telehealth formats.

I’m not a person to talk to a machine or a program, you know. I like to be with people…I don’t see the person I’m talking with, and the reaction is different when you are face-to-face. (A43)

Four participants indicated a belief that face-to-face, in-person contact allowed clinicians and people accessing their service to better read each other's facial and body language cues, which some believed were particularly important for people commencing therapy for the first time.

I guess the face-to-face is probably even better. The counsellor can see our facial expressions, and you can read a bit about a person's facial expressions at times. (A44)

One participant expressed the belief that group formats were not possible online. Another participant's preference for in-person services appeared to be attributable to the clinician's lack of skills with the format, rather than the format itself.

I hated it. It was so impersonal. It was just awkward and awful…It was just terrible because she had noise in the background. Her cat kept coming on the computer and it was so unprofessional. (AB35)

Other reasons for preferring in-person services included discomfort with using the telephone in everyday life (n = 3); the privacy enabled by in-person services conducted outside the family home (n = 1); the opportunity to get out of the house (n = 1); a parent's belief that telehealth services are inappropriate for school-aged children (n = 1); and the advice of a clinician that in person services were more beneficial (n = 1).

#### Preference for telehealth services

Participants who preferred telehealth services or a combination described a range of benefits of telehealth. These predominantly included the convenience (n = 10), for example, being able to access services despite busy work schedules and family responsibilities, and not needing to travel.

I think it was really good for me personally, just because [I’m] pregnant and then having a young toddler already, it was good to just be able to [use] the speaker over the phone or zoom. Having multiple options rather than rushing around and trying to find care for my baby. And then having to worry about leaving the house late and things like that. (A25)

Telehealth services eliminated the need to travel to access services, which was particularly important for those with mobility issues, those who relied on public transport, and those who lived in rural communities.

The clinic is a bit far from my house and I was working during that period so it's a bit far for me to spend two hours on public transport. (A38)

Nine participants talked about feeling more emotionally comfortable and psychologically safer with telehealth as they were able to access services from home.

I felt a bit more comfortable at home with my family. And I had my animals there as well, which they’re kind of my other support network. (A15)

I don’t have to stress myself out about going into an appointment and having to front up to somebody when I’m not feeling really well. I can be at home comfortable on my couch while I’m talking about the things that are difficult for me. It was a much less confronting experience. (AB4)

Three of these participants reported that they or their loved one were more able to be open and honest with telehealth. They said they were better able to focus on the objective of the telehealth session rather than being preoccupied with managing the interpersonal aspects of the interaction.

I think being on the phone was actually easier than him having to go in face-to-face for an appointment. I think for him, it was just that separation, perhaps not feeling as necessarily judged because you don’t have that person in front of you, watching you as you’re talking. He found it really, really good. (AB4)

When I know that I’m having a bit of an emotional day I’d prefer over the phone so that when I start crying I can mute myself and I can just get myself together. (A9)

Six participants highlighted the reduction in waiting times and immediate access to services facilitated by telehealth services, particularly during times of lockdown and for those living in rural and regional areas. Other reasons for preferring telehealth services less frequently cited included enabling access to services in their mother tongue (n = 1); and the ability to ‘multitask’ during telehealth sessions (n = 1). Only three participants overall mentioned encountering occasional technical difficulties with telehealth, none of whom preferred face-to-face service overall.

#### Preference for a combination of service types

Participants who valued a mix of service types (n = 8) suggested that different formats may suit different people at different times. One participant summed it up this way:

On the phone it works quite well for my lifestyle, but face-to-face is nice sometimes too because you get to see them. But yeah, I liked having the blend. (A14)

Table 2 presents a summary of the reasons for preference for either in-person or telehealth services given by participants according to the service mode received. While qualitative research does not lend itself to conclusions about between-group differences, the table shows that themes emerged across users receiving different modes of service. However, those receiving only in-person services reported no reasons for preferring telehealth and those receiving only videoconference or telephone and videoconference services discussed no reasons to prefer in-person services.

**Table 2.** Reasons given for expressed preference.

	Total (*n *= 45)^a^	Service mode received					
		In-person only (*n *= 12)^b^	VC^c^ only (*n *= 4)	Telephone only (*n *= 11)^a^	In-person and telephone (*n *= 9)^a^	In-person + VC^c^ (*n *= 7)^a^	Telephone + VC^c^ (*n *= 2)^a^
Reason for preference for in-person services							
Superior human contact	16	5	-	3	5	2	-
Discomfort with telephone	3	2	-	-	1	-	-
Group formats not compatible with telephone	1	-	-	1	-	-	-
Clinician lack of skill	1	-	-	-	-	1	-
Privacy	1	-	-	-	-	1	-
Opportunity to get out of house	1	1	-	-	-	-	-
Telehealth inappropriate for children	1	1	-	-	-	-	-
Advice of clinician	1	-	-	1	-	-	-
Reason for preference for telehealth services							
Convenience	10	-	1	1	3	3	-
Emotionally comfortable, safer	9	-	2	4	-	-	-
Immediate access	6	-	1	2	1	-	2
‘Multitasking’ during sessions	1	-	-	1		-	-
Access to services in mother tongue	1	-	1	-	-	-	-

^a^ Column totals may >100% as participants reported multiple reasons for their preference. ^b^3 participants receiving in-person services did not provide specific reasons for their preference. ^c^VC: videoconferencing.

## Discussion

In this study, we examined people's experiences of accessing a new mental health service during the COVID-19 pandemic in Victoria, Australia. Approximately half of the participants in our study preferred telehealth or liked it as an option; these participants were more likely to have had direct experience receiving telehealth services, particularly where these had been provided via videoconferencing. Reasons for a preference for telehealth services included beliefs in its convenience and the access it facilitated to necessary services, feeling safer or more comfortable during telehealth-delivered services, and the belief that they could communicate more openly in telehealth services. Conversely, when describing preferences for in-person services, participants emphasised a belief that this mode of service delivery facilitated superior interpersonal communication, was compatible with their personal communication style, and cited their personal discomfort with technology.

Those who provide services have generally assumed people prefer in-person services and that telehealth services should only be offered as an option of last resort.^29^ While our evaluation partners reported from their consultations with service providers that many felt clients mostly preferred in-person services, this did not seem to be borne out by what we heard from all participants in the study.^29^ Our findings that some participants described heightened comfort and sense of safety during telehealth-delivered sessions challenge the assumption of the inherent superiority of interpersonal interactions conducted in-person. Other researchers have similarly noted that some service users find telehealth less confronting than in-person services.^36^ Telehealth delivery of mental health services therefore appears to have potential application beyond pandemic situations or for providing access for people in regional and remote areas, for example, to access services otherwise unavailable or services in different languages. Rather, it appears that many people accessing mental health supports regard telehealth as a legitimate service option. The option of telehealth may appeal to people who are reluctant or unable to access face-to-face services, potentially contributing to narrowing the gap between the number of people who would benefit from mental health support and the number of people currently accessing it.^26^

Current indications suggest that the widespread use and uptake of telehealth related to COVID-19 has brought changes which are likely to be sustained beyond the end of COVID-19 public health restrictions.^3,13^ Despite some ongoing reservations, mental health practitioners express an intention to continue using telehealth for mental health service delivery beyond the end of COVID-19.^12,21^ Our findings suggest that this development is likely to be well-received by many people accessing mental health help as it offers enhanced choice and access exceeding that achievable via in-person services alone. These findings have important implications for future policy and service delivery. In particular, a ‘one size fits all’ approach to service delivery is unlikely to maximise the benefits associated with either in-person or telehealth service delivery models. Individual models of telehealth can be developed that enable the flexible selection of components that reflect the specific needs and preferences of people accessing services. This could include where telehealth service will be accessed from, whether inside or outside of the family home, when services are provided, either within or after usual business hours, whether video functionality will be utilised, and whether and how frequently in-person sessions will augment telehealth via hybrid models of service delivery. Since many people value having at least some in-person interactions with service providers prior to engaging in telehealth,^37^ hybrid models that combine both modes of service delivery may in particular be highly acceptable.

Telehealth delivery of mental health services also provides enhanced opportunities to extend the reach of service providers and help to ensure that resources are more fully and equitably utilised across geographic areas. Telehealth may help to overcome the challenge of matching people who have unique needs to clinicians with requisite skills and attributes living in different geographical areas. Such needs may relate to culture, language, age, gender or additional diagnoses such as autism or intellectual disability. To harness the benefits of telehealth in facilitating increased access and choice, health policies will need to be adjusted to enable services to move from strict state-based geographical approaches to service provision employing rigid criteria related to location.

In our study, acceptability of telehealth appeared to increase with direct experience of telehealth services, with those with direct experience of telehealth more likely to prefer telehealth services in the future. While it is possible that this was at least partly due to people with positive perceptions of telehealth being more likely to receive telehealth services, previous research supports the notion that the acceptability of telehealth services increases with experience.^19,38^ Findings in our small sample suggest that experience of videoconferencing rather than telephone may be especially effective at promoting positive views of telehealth. Further, some participants’ comments about the limitations of telehealth relating to non-verbal communication and incompatibility with group-based formats appear to reflect the receipt of telephone-based services rather than videoconferencing, which can both allow some non-verbal communication and enable group-based sessions. It is unclear whether these participants would express similar beliefs regarding services provided via telehealth formats such as videoconferencing which enable face-to-face and group-based contact. Conversely, some participants experienced the lack of visual input in telephone interactions as a benefit, highlighting the need for individuals to be provided with choice and a flexible trial-and-error approach where people lack experience with telehealth. A small number of participants indicated that they were deterred from engaging with telehealth either due to the lack of skill or the advice of service providers. Such observations are unsurprising, given the speed with which providers pivoted to telehealth delivery of mental health services at the outset of COVID-19, in many cases with minimal or no training to support its uptake.^25^ Further research could explore the impact of service provider skills with, and attitudes towards, telehealth on the experiences and preferences of those accessing telehealth services. These findings also indicate that ongoing support and training will be required to ensure clinicians have the necessary strong professional skills required to deliver telehealth services that not only are effective, but that strengthen the beliefs of those accessing support in its suitability for them as they commence telehealth services for the first time.^19^ Additional research is required to elucidate the specific competencies required of clinicians for quality telehealth service delivery; such information will help organisations provide the necessary support and training to clinicians and help to support positive service user experiences of telehealth and, ultimately, enhanced outcomes.

Given increasing evidence of its value, telehealth should be a crucial consideration in the development of future mental health service models. Rather than viewing telehealth as an addition to or needing to fit within existing models, therapeutic environments should be developed to foster good telehealth arrangements and offer support to both those who access services and those who provide them to feel safe and comfortable with telehealth.

### Limitations

Several limitations to this study should be considered. The participants were drawn from a population of people with mental health concerns who did not require acute care, but whose needs were also likely to be too complex to be adequately addressed by a general practitioner or minimal counselling support, the so-called ‘Missing Middle’.^29^ Whilst it is a strength of this study that we were able to gain insights into the perceptions of this thus far underexplored group, further research is needed to understand to what degree the current findings apply to other diverse groups. Further, we sought to understand participants’ experiences of telephone or videoconferencing for service provision using interviews that were conducted using these same mediums. Although the current study was strengthened by purposive sampling, which ensured inclusion of a large and varied sample of participants encompassing a wide range of satisfaction with their overall services, from very negative to very positive, it is possible that those with the most negative attitudes towards telehealth may not have volunteered for the study. Finally, experiences and attitudes towards telehealth were not the primary focus of the larger study, so data saturation was not based on telehealth-related categories. Even so, we were able to capture a range of perspectives on telehealth and in-person services which provided sufficient coverage of the relevant issues. However, further primary research in which purposive sampling is based on telehealth use and experience and discussion focuses on the mode of service delivery in unfamiliar services will add to depth of understanding of these issues.

## Conclusions

The COVID-19 pandemic experience has profoundly shifted the ways in which health services, including mental health, are delivered across the world. Service providers in many countries are now more clearly able to determine the potential of telehealth for expanding choice and equalising access across vast geographical distances, without the fear of sacrificing quality of care or satisfaction. Whilst in-person services will always have an important and fundamental role in models of care, the current findings add to the growing evidence that telehealth should be included in flexible suites of service delivery options that ensure person-centred care.
